# Aberrant intrinsic brain activities in functional gastrointestinal disorders revealed by seed-based d mapping with permutation of subject images

**DOI:** 10.3389/fnins.2024.1452216

**Published:** 2024-11-15

**Authors:** Yibin Shuai, Benhong Wang, Xiaomei Zhang, Zhongxia Shen, Shengbo Han, Cong Zhou

**Affiliations:** ^1^Sleep Medical Center, Huzhou Third Municipal Hospital, The Affiliated Hospital of Huzhou University, Huzhou, China; ^2^School of Clinical Medicine, Jining Medical University, Jining, China; ^3^School of Mental Health, Jining Medical University, Jining, China; ^4^Department of Psychology, Affiliated Hospital of Jining Medical University, Jining, China

**Keywords:** functional gastrointestinal disorders, resting-state fMRI, irritable bowel syndrome, functional dyspepsia, functional constipation, seed-based d mapping

## Abstract

Functional gastrointestinal disorders (FGIDs) are characterized by complex interactions between the gut and brain, leading to altered brain function and symptom manifestation. We used neuroimaging meta-analytic techniques in order to analyze the correlation between FGIDs and aberrant brain activity. A systematic review was performed to ascertain resting-state functional magnetic resonance imaging (rs-fMRI) studies examining brain function in FGIDs. Pooled meta-analyses by seed-based d mapping with permutation of subject images (SDM-PSI) were performed to assess variations in regional brain activity, and sensitivity analyses were applied to evaluate the robustness of findings. Meta-regression analyses were then carried out to examine possible links between demographic factors and neuroimaging changes. Our meta-analysis revealed significant changes in regional brain activities among FGIDs patients compared to healthy controls (HC). Increased brain activation was observed in several regions including the postcentral gyrus, calcarine fissure/surrounding cortex, superior frontal gyrus, and insula, while decreased activity was noted in the left posterior cingulate gyrus, right median cingulate/paracingulate gyri, and the left caudate nucleus. Furthermore, meta-regression analyses indicated negative associations between disease duration and alterations in specific brain regions. These findings underscored the intricate interplay between gut dysfunction and aberrant brain activity in FGIDs. Early intervention and multidisciplinary approaches addressing both gastrointestinal symptoms and associated emotional distress are crucial for improving the quality of life of the patients.

## Introduction

Functional gastrointestinal disorders (FGIDs) encompass a cluster of chronic conditions affecting the alimentary system, characterized by recurrent symptoms without identifiable structural or biochemical abnormalities. Common FGIDs include irritable bowel syndrome (IBS), functional dyspepsia (FD), and functional constipation (FC) (Black et al., 2020). According to global epidemiological studies, FGIDs affect approximately 10–20% of the world’s population (Suares and Ford, 2011; Lovell and Ford, 2012; Aro et al., 2015; Kano et al., 2018; Sperber et al., 2021), leading to substantial healthcare utilization, impaired quality of life, and significant economic burden. IBS presents with abdominal pain and altered bowel habits, FC is characterized by infrequent bowel movements and difficulty in defecation, while FD involves persistent upper abdominal pain or discomfort without an identifiable organic cause. These disorders manifest with symptoms such as abdominal pain, bloating, altered bowel habits, and indigestion, often resulting in reduced productivity and psychological distress among patients (Black et al., 2020; Sperber et al., 2021). Understanding the prevalence and impact of FGIDs is crucial for effective management and public health interventions to alleviate the burden on individuals and healthcare systems.

The FGIDs are closely associated with psychological and emotional states, often overlapping with conditions such as depression and anxiety. Psychological factors such as stress and negative life events can influence gut motility, visceral sensitivity, and immune function, contributing to the onset and exacerbation of FGIDs (Black et al., 2020). Research has shown that individuals with FGIDs frequently experience comorbid psychiatric conditions. For example, a longitudinal study by Aro et al. (2015) found that anxiety was related to new-found indigestion in the population of Sweden, suggesting a potential causal relationship between psychological distress and gastrointestinal symptoms. Similarly, a meta-analysis by Lovell and Ford (2012) revealed a higher prevalence of anxiety and depression in patients with IBS compared to the general population. The gut-brain axis is recognized as pivotal in the development of FGIDs, facilitating bidirectional communication between the central nervous system and the enteric nervous system (Mayer et al., 2022). This axis plays a critical role in modulating both gastrointestinal function and emotional responses (Mayer et al., 2022; Gong et al., 2023). Dysregulation of the gut-brain axis, characterized by altered neurotransmitter signaling, immune activation, and changes in gut microbiota composition, has been implicated in the pathophysiology of FGIDs (Mayer et al., 2015a; Mayer et al., 2015b). For example, stress-induced alterations in gut microbiota composition can impact gut permeability and immune function, contributing to intestinal inflammation and visceral hypersensitivity observed in FGIDs (Mayer et al., 2015b), which might underlie the patients’ gastrointestinal symptoms.

Recent advances in neuroimaging technology have facilitated the exploration of brain function through non-invasive methods, with resting-state functional magnetic resonance imaging (rs-fMRI) emerging as a prominent technique. Among this, regional homogeneity (ReHo), amplitude of low-frequency fluctuations (ALFF), and fractional ALFF (fALFF) are commonly utilized approaches for exhibiting local spontaneous activity in rs-fMRI data (Zang et al., 2015). Each of these metrics offers unique insights into regional spontaneous brain activities, collectively enhancing our understanding of brain function in health and disease (Zang et al., 2015; Salvia et al., 2019). Numerous neuroimaging studies have revealed abnormal brain structure and function in individuals with FGIDs, emphasizing the significance of the gut-brain axis in the development of these disorders (Kano et al., 2018). However, current findings are inconsistent, possibly due to the heterogeneity in samples and imaging methodologies.

Meta-analysis serves as a robust method to consolidate neuroimaging observations from diverse researches, offering an all-sided synthesis of regional alterations. This method not only addresses discrepancies among neuroimaging studies but also distinguishes between spurious results and reproducible findings. By aggregating data across studies, meta-analysis provides a unified perspective and facilitates the integration of vast amounts of information (Muller et al., 2018). Among these techniques, the seed-based d mapping with permutation of subject images (SDM-PSI) is a notable progressive statistical method for coordinate-based meta-analysis (CBMA) (Radua et al., 2012). SDM-PSI allows for the objective and quantitative integration of diverse neuroimaging findings. One former meta-analysis investigated irregular local spontaneous functional activity during resting states in IBS patients, identifying transformations in brain regions associated with emotional management and somatic sensation (Su et al., 2022). Similarly, a systematic review by Yu et al. (2022) highlighted altered resting brain functions in IBS patients, implicating disruptions in neural networks involved in pain modulation and emotional regulation. Furthermore, another meta-analysis examined alterations in default mode network functionality and gray matter structure in IBS patients, emphasizing abnormalities in brain regions responsible pain perception, transmission and interpretation (Zhao et al., 2023). Besides, a CBMA found changes in brain regions responsible for visceral sensation, pain modulation, and emotion regulation in FD patients (Mao et al., 2023). One recent review indicated that FC is linked to changes in brain function and structure, especially in regions and networks related to emotion regulation, motor control, somatic sensation, and self-referential processing (Feng et al., 2023). These studies provided valuable insights into the neuropathological characteristics of FGIDs and underscored the importance of understanding the brain-gut axis in these disorders. However, these studies conducted meta-analysis focusing on a specific disorder, which might contain certain limitations.

This present study aims to enhance CBMA across various FGIDs, focusing on consolidating regional aberrations in brain activity utilizing ReHo, ALFF, and fALFF metrics. Additionally, with the approach of meta-regression, we seek to explore the latent influence of demographics and clinical parameters on brain functions, such as age and disease duration. We expect these findings will provide valuable insights to advance the diagnosis and treatment of FGIDs in clinical practice. Based on previous research findings, we hypothesize that FGIDs patients may manifest altered brain activations in regions associated with emotion regulation, pain perception, and sensory processing such as the frontal gyrus and insula.

## Methods

### Literature search strategy

The protocol for this CBMA was duly registered with PROSPERO (registration number: CRD42024536106; accessible at http://www.crd.york.ac.uk/PROSPERO), underscoring our commitment to methodological rigor and transparency. The current meta-analysis adhered strictly to the Preferred Reporting Items for Systematic Reviews and Meta-Analyses (PRISMA) guidelines (Liberati et al., 2009; Moher et al., 2009a,b; Page et al., 2021) as well as the rules for neuroimaging meta-analysis (Muller et al., 2018) to ensure transparency and reliability in our methods. Relevant literature was systematically sourced through comprehensive searches of the PubMed, Web of Science, and Cochrane Library databases, encompassing publications up to January 31, 2024. The search strategy involved keywords including (“functional gastrointestinal disorders” or “FGIDs” or “irritable bowel syndrome” or “IBS” or “functional constipation” or “functional dyspepsia”) in conjunction with [(“resting state” or “resting-state” or “at rest” or “resting”) or (“amplitude of low frequency fluctuation” or “fractional amplitude of low frequency fluctuation” or “ALFF” or “fALFF”) or (“regional homogeneity” or “ReHo” or “local connectivity” or “coherence”)]. Moreover, for the purpose of preventing oversight, we conducted a manual examination of the reference lists of approved studies and correlated reviews.

### Study selection

The criteria for the selection of studies were delineated as follows: (1) Studies comparing ReHo, ALFF, or fALFF values between patients with FGIDs and HC through whole-brain analyses were included. (2) Results needed to be provided in either Talairach or Montreal Neurological Institute (MNI) coordinates. (3) Utilization of a significance threshold; (4) publication in peer-reviewed journals and composed in the English language. The criteria for exclusion were as below: (1) meta-analyses, reviews, and case reports were excluded. (2) Studies that did not include direct between-group comparisons were excluded. (3) Studies were excluded if the peak coordinates or parametric maps were inaccessible.

### Quality assessment and data extraction

The process of quality assessment and data extraction was executed by two authors (S.Y. and W.B.) respectively. They performed literature searches, evaluated the quality of retrieved articles, and extracted and cross-validated data from eligible articles. Additionally, both authors independently assessed the quality of the final studies in accordance with guidelines for neuroimaging meta-analyses (Muller et al., 2018). We documented the following parameters: lead author, sample size, participant characteristics (such as age and gender), criteria for diagnosis, illness duration, imaging protocols, methods of data processing, and the statistical thresholds applied in individual studies.

### Meta-analysis

We performed meta-analyses using SDM software (Radua and Mataix-Cols, 2009; Albajes-Eizagirre et al., 2019) to investigate discrepancies in local brain activation between FGIDs patients and HC. The SDM-PSI technique, known for its robust statistical approach, utilizes peak coordinates to evaluate disparities in cerebral activity (Radua et al., 2012). The detailed procedures of SDM-PSI, outlined extensively elsewhere (Duan et al., 2022; Liu et al., 2022; Wang et al., 2022), and are succinctly summarized as follows: (1) the software generated effect-size maps illustrating differences in regional activities between patients and HC for each study, based on peak coordinates of effects and associated statistics, such as *t*-statistics. Significant cluster *Z-* or *p*-values were transformed to t-statistics using the SDM online converter. (2) Peak coordinates for each study were reconstructed utilizing a standard MNI map of effect size for group differences in neuroimaging, employing an anisotropic Gaussian kernel (Radua et al., 2014). (3) A comprehensive meta-analysis was performed to generate a mean map through voxel-wise computation of the random-effects mean of the study maps.

Following the methodology established by Radua et al. (2012), we adopted *p* = 0.005 in SDM-PSI analyses. Furthermore, we employed a peak height threshold of *Z* = 1.00 and a cluster size threshold of 10 voxels to ensure robustness in our findings.

### Sensitivity analyses

We then conducted sensitivity analyses to assess the repeatability of our findings. If a particular brain region consistently demonstrated significance across the majority or all combinations of studies during the jackknife sensitivity analysis, it was considered highly replicable (Radua and Mataix-Cols, 2009).

### Subgroup meta-analyses

We conducted subgroup meta-analyses focusing exclusively on each subtype of the FGIDs (IBS, FD, and FC).

### Meta-regression analyses

Meta-regression analyses were performed in each patient group to explore potential demographic variables on neuroimaging changes. We used *p* < 0.0005 to serve as a threshold for significance (Radua and Mataix-Cols, 2009). We only considered brain regions identified in the main effect.

## Results

### Sample characteristics of included studies

**Figure 1:**
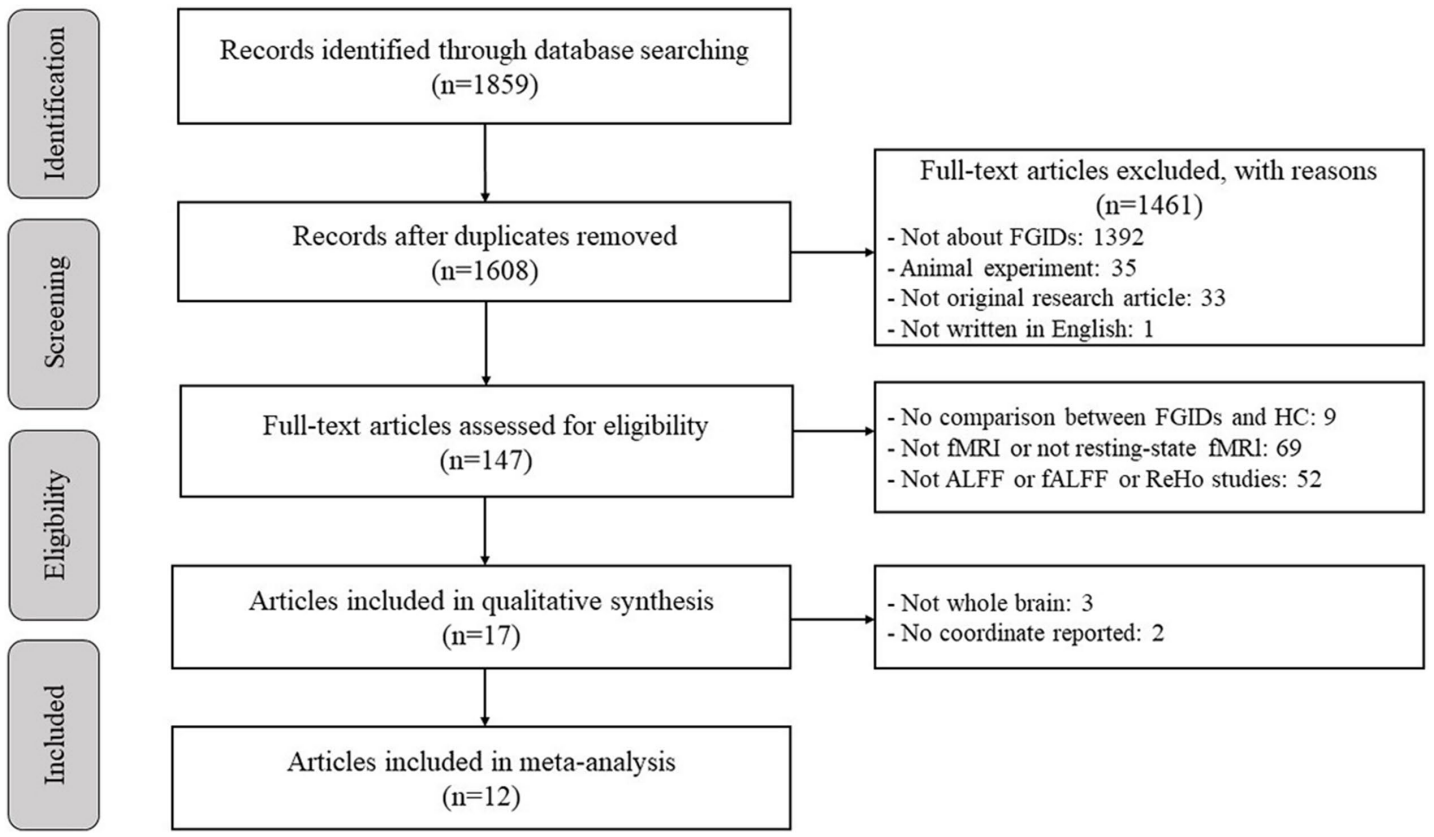
Flow diagram for the identification and exclusion of studies.

Figure 1 depicts a flow diagram outlining the process of including studies related to FGIDs. Table 1 summarizes the demographic characteristics and neuroimaging methodologies utilized in each subgroup corresponding to the studied diseases. We identified 1895 studies under the search strategy, with 12 fitting the predefined inclusion criteria (Zhou et al., 2013; Nan et al., 2014; Ke et al., 2015; Ma et al., 2015; Qi et al., 2016; Lee et al., 2018; Jin et al., 2019; Qi et al., 2020; Ao et al., 2021; Chen et al., 2021; Li et al., 2021; Cai et al., 2024). Notably, the study by Qi et al. (2020) included two subgroups of FD, while the study by Li et al. (2021) encompassed two subgroups of functional constipation FC patients. Additionally, Chen et al. (2021) conducted both ALFF and ReHo analyses. The final sample comprised 463 patients and 469 healthy controls, with 99 coordinates extracted from 15 datasets.

**Table 1:** Demographic and clinical characteristics and the neuroimaging approaches of the participants in the studies included in the meta-analyses.

	Sample size (female)		Mean age (SD)		Diagnostic criteria	Duration (month)	Scanner	Indices	Statistical threshold	Number of coordinates
	Patients	HC	Patients	HC						
IBS studies										
Ke et al. (2015)	31 (6)	32 (7)	29.2	27.5	Rome III criteria	32.7	3.0T	ReHo	*p* < 0.05, FDR corrected	19
Ma et al. (2015)	21 (7)	21 (10)	41.8	35.9	Rome III criteria	59.0	1.5T	ALFF	*P* < 0.05, Alphasim corrected	8
Qi et al. (2016)	30 (6)	31 (7)	28.9	26.9	Rome III criteria	20.4	3.0T	ALFF	*P* < 0.05, Alphasim corrected	11
Ao et al. (2021)	13 (5)	14 (6)	32.2	29.1	Rome III criteria	16.6	3.0T	fALFF	*p* < 0.005, uncorrected	3
Chen et al. (2021)	36 (20)	36 (26)	34.4	31.7	Rome III criteria	19.1	3.0T	ALFF	*P* < 0.05, FWE corrected	9
Chen et al. (2021)	36 (20)	36 (26)	34.4	31.7	Rome III criteria	19.1	3.0T	ReHo	*P* < 0.05, FWE corrected	6
FD studies										
Zhou et al. (2013)	29 (16)	16 (10)	22.4	21.9	Rome III criteria	33.7	3.0T	fALFF	*P* < 0.05, TFCE corrected	5
Nan et al. (2014)	40 (29)	20 (12)	22.3	22.1	Rome III criteria	37.2	3.0T	ReHo	*P* < 0.05, FDR corrected	7
Lee et al. (2018)	12 (7)	14 (9)	46.5	45.8	Rome III criteria	156.0	3.0T	ALFF	*P* < 0.05, FWE corrected	6
Qi et al. (2020)	18 (7)	22 (13)	43.8	41.4	Rome III criteria	48.9	3.0T	ALFF	*P* < 0.05, RFT corrected	7
Qi et al. (2020)	13 (6)	22 (13)	41.8	41.4	Rome III criteria	54.0	3.0T	ALFF	*P* < 0.05, RFT corrected	6
FC studies										
Jin et al. (2019)	46 (34)	53 (35)	40.0	40.8	Rome IV criteria	120.8	1.5T	ALFF	*P* < 0.05, FWE corrected	1
Li et al. (2021)	37 (29)	42 (25)	40.9	40.6	Rome IV criteria	105.6	1.5T	fALFF	*P* < 0.05, FWE corrected	5
Li et al. (2021)	28 (19)	42 (25)	43.0	40.6	Rome IV criteria	112.8	1.5T	fALFF	*P* < 0.05, FWE corrected	2
Cai et al. (2024)	73 (53)	68 (45)	51.1	53.2	Rome IV criteria	N/A	3.0T	ReHo	*P* < 0.05, FWE corrected	3

ALFF, amplitude of low-frequency fluctuation; fALFF, fractional amplitude of low-frequency fluctuation; FC, functional constipation; FD, functional dyspepsia; FDR, false discovery rate; FWE, family-wise error; HC, healthy controls; IBS, irritable bowel syndrome; N/A, not available; ReHo, regional homogeneity; RFT, random-field theory; T, Tesla; TFCE, threshold-free cluster enhancement.

### Meta-analyses findings

**Figure 2:**
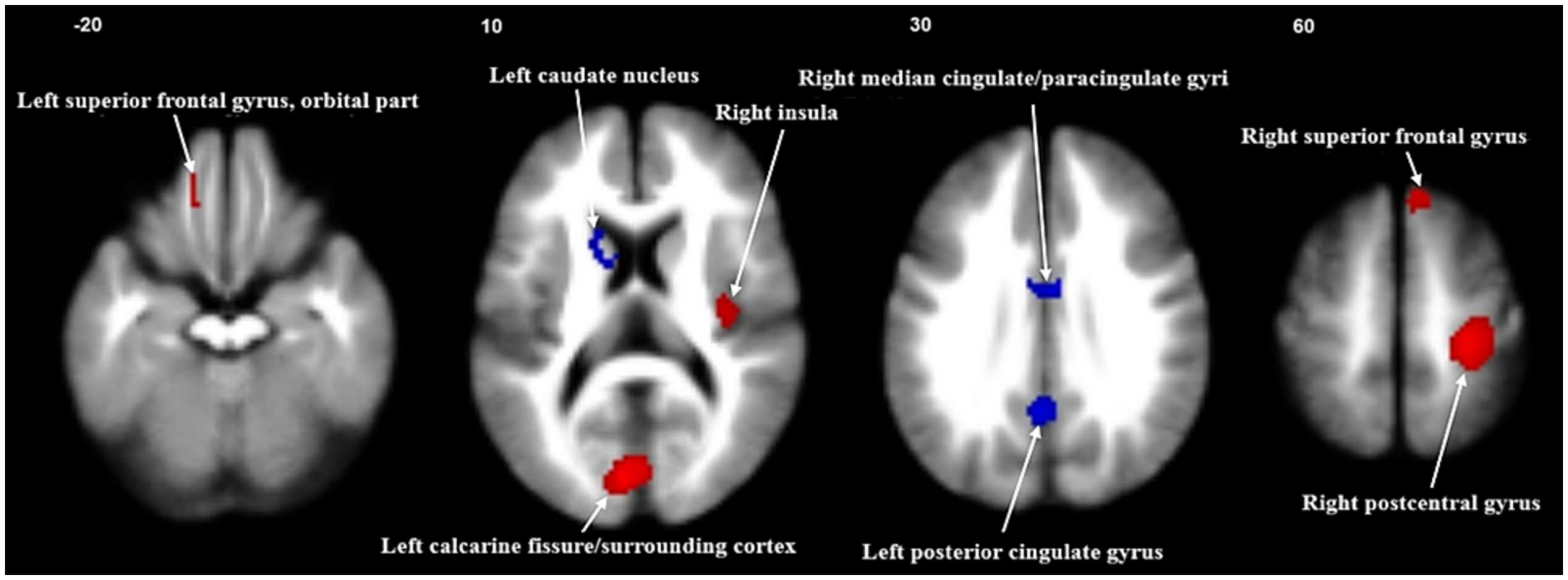
Meta-analysis of regional abnormal resting-state brain activities in functional gastrointestinal disorders. Significant clusters are overlaid on MRIcron template for Windows for display purposes only.

The primary meta-analysis unveiled that FGIDs patients displayed significantly heightened brain activities across five clusters, notably encompassing the right postcentral gyrus, left calcarine fissure/surrounding cortex, right superior frontal gyrus (medial), right insula, and the left superior frontal gyrus (orbital part). Additionally, three clusters exhibited decreased brain activities, including the left posterior cingulate gyrus, right median cingulate/paracingulate gyri, and the left caudate nucleus. These results are visually shown in Figure 2 and thoroughly summarized in Table 2.

**Table 2:** Regional brain spontaneous activity changes in functional gastrointestinal disorders.

Regions	Maximum					Cluster		Jackknife sensitivity analysis
	MNI coordinates			SDM Value	*P*	Number of voxels*	Breakdown (number of voxels)	
	*X*	*Y*	*Z*					
FGIDs > HC								
Right postcentral gyrus, BA 4	24	−30	62	1.946	0.000098050	631	Right precentral gyrus, BA 6 (186) Right postcentral gyrus, BA 4 (118) Right precentral gyrus, BA 4 (112) Right postcentral gyrus, BA 3 (101) Right postcentral gyrus, BA 6 (31) Corpus callosum (25) Right hand superior U tract (24) Right postcentral gyrus, BA 2 (14) Right precentral gyrus, BA 3 (6) Right precentral gyrus (5) Right postcentral gyrus (5) Right superior longitudinal fasciculus II (4)	15/15
Left calcarine fissure/surrounding cortex, BA 18	−4	−82	12	1.948	0.000098050	533	Left calcarine fissure/surrounding cortex, BA 17 (150) Corpus callosum (127) Right calcarine fissure/surrounding cortex, BA 17 (65) Left lingual gyrus, BA 17 (48) Left calcarine fissure/surrounding cortex, BA 18 (42) Left cuneus cortex, BA 18 (38) Right inferior network, inferior longitudinal fasciculus (25) Right lingual gyrus, BA 17 (22) Left calcarine fissure/surrounding cortex (11) Right calcarine fissure/surrounding cortex, BA 18 (2) Left inferior network, inferior longitudinal fasciculus (2) Left lingual gyrus (1)	13/15
Right superior frontal gyrus, medial, BA 8	4	26	62	1.558	0.001661777	92	Right superior frontal gyrus, medial, BA 8 (83) Right supplementary motor area, BA 8 (7) Right superior frontal gyrus, medial (1) Right superior frontal gyrus, dorsolateral, BA 8 (1)	14/15
Right insula, BA 48	36	−14	12	1.464	0.002915859	95	Right insula, BA 48 (92) Right rolandic operculum, BA 48 (2) Right fronto-insular tract 5 (1)	12/15
Left superior frontal gyrus, orbital part, BA 11	−14	34	−20	1.588	0.001388252	35	Left superior frontal gyrus, orbital part, BA 11 (20) Corpus callosum (8) Left striatum (3) Left gyrus rectus, BA 11 (2) Left inferior network, uncinate fasciculus (1) Left superior frontal gyrus, orbital part (1)	13/15
FGIDs > HC								
Left posterior cingulate gyrus, BA 23	−2	−54	26	−1.854	0.000438690	295	Left precuneus, BA 23 (68) Left posterior cingulate gyrus, BA 23 (39) Right precuneus, BA 23 (36) Left precuneus (33) Left posterior cingulate gyrus (21) Left precuneus, BA 30 (21) Left posterior cingulate gyrus, BA 30 (17) Right posterior cingulate gyrus, BA 23 (11) Left median network, cingulum (7) Right median cingulate/paracingulate gyri, BA 23 (7) Right posterior cingulate gyrus, BA 30 (7) Right precuneus (7) Right precuneus, BA 30 (6) Right posterior cingulate gyrus (2) Left posterior cingulate gyrus, BA 26 (2) Right posterior cingulate gyrus, BA 26 (1) (undefined) (8) (undefined), BA 30 (2)	13/15
Right median cingulate/paracingulate gyri, BA 24	8	2	42	−2.019	0.000154853	244	Right median cingulate/paracingulate gyri, BA 24 (43) Right median cingulate/paracingulate gyri, BA 32 (39) Right median cingulate/paracingulate gyri (25) Left median cingulate/paracingulate gyri, BA 24 (19) Right median network, cingulum (14) Right supplementary motor area, BA 24 (12) Corpus callosum (12) Right median cingulate/paracingulate gyri, BA 23 (11) Left median network, cingulum (11) Right supplementary motor area, BA 32 (10) Right supplementary motor area (10) Left supplementary motor area, BA 32 (8) Left median cingulate/paracingulate gyri, BA 23 (7) Left supplementary motor area (6) Left median cingulate/paracingulate gyri (5) Left anterior cingulate/paracingulate gyri (4) Left supplementary motor area, BA 24 (2) Right supplementary motor area, BA 6 (2) (undefined) (4)	15/15
Left caudate nucleus	−12	16	14	−1.749	0.000856698	80	Left anterior thalamic projections (51) Left caudate nucleus (19) Left striatum (7) Corpus callosum (1) Left caudate nucleus, BA 25 (1) (undefined) (1)	14/15

^*^All voxels with *P* < 0.005 uncorrected. BA, Brodmann area; FGIDs, functional gastrointestinal disorders; HC, healthy controls; MNI, Montreal Neurological Institute; SDM, seed‐based d mapping.

### Sensitivity analysis

The sensitivity analysis, conducted through whole-brain jackknife analyses, demonstrated high reproducibility of the results. Specifically, consistently significant findings across all dataset combinations included increased brain activity in the right postcentral gyrus and reduced activity in the right median cingulate/paracingulate gyri. Likewise, heightened brain activity in the right superior frontal gyrus (medial) and reduced brain activity in the left caudate nucleus remained consistently significant across all but one dataset combination. Additionally, the remaining clusters retained significance in all but three dataset combinations. Detailed information can be found in Table 2.

### Subgroup analysis

Despite the inclusion of a sample size below the recommended minimum of 10 datasets for SDM meta-analyses, we conducted an exploratory analysis of different subtypes of FGIDs. Detailed results of the subtypes of FGIDs are provided in Supplementary Table S1. The findings from subgroups partially aligned with the pooled meta-analysis.

### Meta-regression analysis

Meta-regression analysis unveiled a negative correlation between the duration of FGIDs in patients and brain activity changes, particularly within the right median cingulate/paracingulate gyri and the left caudate nucleus (Table 3).

**Table 3:** Negative correlations between duration and regional brain activity alterations in FGIDs patients revealed by meta‐regression analysis.

		MNI coordinates					
Factor	Anatomic label	*X*	*Y*	*Z*	SDM Value	*P*	Number of voxels
Duration	Right median cingulate/paracingulate gyri, BA 24	6	4	42	−3.982	~0	758
	Left caudate nucleus	−14	16	12	−2.764	0.000294149	18

FGIDs, functional gastrointestinal disorders; MNI, Montreal Neurological Institute; SDM, seed-based d mapping.

## Discussion

Consistent with prior studies, our study revealed significantly changed regional brain functions in several regions including increased brain activities in the postcentral gyrus, calcarine fissure/surrounding cortex, superior frontal gyrus, and insula, as well as lower activation in the left posterior cingulate gyrus, right median cingulate/paracingulate gyri, and the left caudate nucleus. These findings reflected the complex interplay between the gut and brain in FGIDs. The observed negative association between the duration of FGIDs and alterations in specific brain regions suggested potential disease progression effects on neural function, highlighting the dynamic nature of gut-brain interactions in FGIDs patients.

The identified alterations in brain function observed in various regions implicated in sensory processing, emotional regulation, and cognitive control provide valuable insights into the pathophysiology of FGIDs (Yu et al., 2022; Mao et al., 2023). The increased brain activities detected in the postcentral gyrus, calcarine fissure/surrounding cortex, superior frontal gyrus, and insula are indicative of heightened sensory processing and visceral sensation perception (Hong et al., 2013). The postcentral gyrus, known for its role in somatosensory processing, may contribute to the heightened perception of visceral pain commonly reported by FGIDs patients (Dong et al., 2022). Similarly, the increased activity in the insula, a key region involved in interoception and emotional processing, suggests dysregulated visceral sensation and emotional responses in FGIDs patients (Zhang et al., 2023). Notably, one previous fMRI study demonstrated that major depressive disorder (MDD) patients with gastrointestinal symptoms showed increased functional asymmetry than those without gastrointestinal symptoms (Fu et al., 2021), this suggests an imbalance in the regulation of brain-gastrointestinal function, emphasizing the involvement of the frontal gyrus in FGIDs.

On the other hand, decreased activation in regions such as the left posterior cingulate gyrus, right median cingulate/paracingulate gyri, and the left caudate nucleus underscored the multifaceted nature of FGIDs (Mayer et al., 2015a). The left posterior cingulate gyrus, known for its involvement in self-awareness, cognition, and emotion regulation, exhibits reduced activity in FGIDs patients compared to healthy controls (Labus et al., 2013). This diminishment in activity might reflect abnormalities in emotional regulation and self-awareness commonly observed in FGIDs patients, which are often linked to anxiety and depression. Additionally, the posterior cingulate gyrus is implicated in pain processing, suggesting its involvement in the altered perception of pain experienced by FGIDs patients (Hong et al., 2014). The cingulate cortex plays crucial roles in emotional regulation and autonomic nervous system function (Berman et al., 2008). The decreased activation observed in right median cingulate/paracingulate gyri might contribute to the emotional dysregulation and autonomic dysfunction often observed in FGIDs patients. Prospective studies have highlighted heightened stress responses and autonomic nervous system dysfunction in FGIDs patients, aligning with the observed reduction in activity in these brain regions (Kano et al., 2018). The caudate nucleus, associated with motor control and reward processing, exhibits decreased activation in IBS patients (Weng et al., 2017). This reduction in activity may be related to the motor dysfunction and altered reward processing observed in FGIDs patients, including changes in gastrointestinal motility and appetite regulation (Kano et al., 2018). It is noteworthy that these brain regions exhibiting aberrant intrinsic brain activities were commonly implicated in emotional disorders such as anxiety and depression (Elsenbruch et al., 2010; Labus et al., 2013), providing microscopic evidence for the frequent comorbidity of FGIDs with mood disturbances. This indicated the intricate interplay between gut-brain interactions and emotional regulation in FGIDs, highlighting the need for comprehensive therapeutic interventions addressing both gastrointestinal symptoms and associated emotional distress. Taken together, the identified alterations in brain activation patterns highlighted the widespread impact of gut dysfunction on brain function in FGIDs. These findings provided insights for further research to elucidate the precise mechanisms underlying these alterations and seek potential interventions targeting these brain regions to relieve symptoms and improve quality of life in FGIDs patients. We propose that future treatments for FGIDs might benefit from targeted interventions aimed at the brain regions implicated in our study, potentially through neuromodulation therapies or cognitive-behavioral strategies.

The gut-brain axis serves as a crucial interface between the gastrointestinal system and the central nervous system, playing a pivotal role in the pathophysiology of FGIDs (Mayer et al., 2022). In line with the predictive coding theory, our findings of altered brain activity in FGIDs may reflect disruptions in the brain’s predictive mechanisms, particularly in processing interoceptive signals from the gut. Dysfunctional gut-brain communication, influenced by factors such as gut microbiota, neuroendocrine signaling, and immune responses, contributes to the onset, progression, and perpetuation of FGIDs symptoms (Mayer et al., 2015a). Reflecting in neuroimaging, our findings demonstrated the significant alterations in brain function observed in FGID patients, highlighted the multifaceted influence of the gut-brain axis on neural processing, and indicated a disrupted homeostatic balance between the gut and brain (Al Omran and Aziz, 2014). In addition, a compelling correlation emerged between the duration of FGIDs and specific alterations in brain regions, suggesting a progressive impact of the disease on neural function. Therefore, it emphasizes the importance of implementing effective treatments and interventions for FGIDs as early as possible. Moreover, in contrast to previous studies, this research conducted an analysis by amalgamating all FGIDs and solely focusing on local brain functional metrics based on ReHo, ALFF, and fALFF, without incorporating other imaging modalities such as positron emission computed tomography (PET) and functional connectivity metrics of fMRI. Subgroup analyses concerning different disorders revealed that FGIDs constitute a complex spectrum of conditions, with IBS, FD, and FC each exhibiting distinct neurobiological mechanisms. However, these findings warrant cautious interpretation given the limited sample size.

The study acknowledges several limitations that should be taken into consideration. Firstly, the heterogeneous nature of data acquisition parameters and clinical variables across the included studies introduced potential biases not fully addressed by statistical methods alone. Secondly, the absence of longitudinal studies in both our meta-analysis and the literature reviewed herein restricted our ability to explore the dynamicity and reversibility of neural activities associated with FGIDs. Longitudinal studies and task-based functional MRI studies are crucial for elucidating the temporal dynamics of brain functions in FGIDs. These studies are especially crucial in examining the temporal interplay between brain activity and the progression of FGID symptoms. Future studies might usefully adopt methodologies that either track these changes longitudinally or involve the presentation of controlled gut-related stimuli to participants. Thirdly, our meta-analysis exclusively focused on changes in resting-state regional spontaneous brain activity in FGIDs, neglecting other valuable aspects such as functional connectivity, graph theory, independent component analysis (ICA), and task-based fMRI studies. Incorporating these methodologies in future investigations could offer a more comprehensive understanding of the functional patterns associated with FGIDs. Lastly, methodological constraints precluded direct comparisons between different subtypes of FGIDs in this study. Future research efforts should strive to overcome these limitations through advancements in analytical techniques.

## Conclusion

In conclusion, our study shed light on the intricate relationship between FGIDs and aberrant brain activity, providing valuable insights into the underlying pathophysiology of these conditions. Our findings revealed significant regional abnormalities in brain activities, implicating areas involved in sensory processing, emotional regulation, and cognitive control. Notably, heightened brain activity in sensory processing regions and reduced activation in areas associated with emotional regulation highlighted the complex interplay between gut dysfunction and brain function in FGIDs.
