# Supplementary material for: Aberrant intrinsic brain activities in functional gastrointestinal disorders revealed by seed-based d mapping with permutation of subject images
Source: Front Neurosci. 2024 Nov 15;18:1452216. doi: 10.3389/fnins.2024.1452216 (PMC11604809; doi:10.3389/fnins.2024.1452216)
Supplement: Supplementary file 1 [file Table_1.doc]

**Supplementary Material**

**Table S1** Regional brain spontaneous activity changes in different subtypes of functional gastrointestinal disorders

| Regions | MNI coordinates  X,Y,Z | SDM Value | *P* | Number of voxels* |
| --- | --- | --- | --- | --- |
| **IBS** |  |  |  |  |
| IBS > HC |  |  |  |  |
| Left calcarine fissure / surrounding cortex, BA 17 | -4,-82,10 | 3.060 | ~0 | 2091 |
| Right precentral gyrus, BA 4 | 42,-24,58 | 2.582 | ~0 | 1560 |
| Right superior frontal gyrus, medial | 2,28,60 | 1.885 | 0.000258029 | 189 |
| IBS < HC |  |  |  |  |
| Left anterior cingulate / paracingulate gyri, BA 24 | -2,28,26 | -1.813 | 0.000510931 | 1002 |
| Right gyrus rectus, BA 11 | 2,42,-20 | -1.677 | 0.001217961 | 374 |
| Left posterior cingulate gyrus, BA 23 | -4,-54,28 | -1.906 | 0.000356078 | 244 |
| Right superior longitudinal fasciculus III | 48,-40,34 | -1.761 | 0.000655413 | 96 |
| Left superior frontal gyrus, medial, BA 9 | -6,48,38 | -1.623 | 0.001924992 | 33 |
| Right middle frontal gyrus, BA 45 | 40,46,20 | -1.517 | 0.003473222 | 14 |
| **FD** |  |  |  |  |
| FD > HC |  |  |  |  |
| Right postcentral gyrus, BA 3 | 32,-32,58 | 1.921 | 0.000319958 | 944 |
| Left superior frontal gyrus, medial | 2,36,36 | 1.633 | 0.002183020 | 635 |
| Right insula, BA 48 | 36,-10,18 | 1.576 | 0.003168762 | 309 |
| Corpus callosum | -12,-26,0 | 1.600 | 0.002657831 | 182 |
| FD < HC |  |  |  |  |
| Left supplementary motor area, BA 6 | -4,-12,64 | -1.206 | 0.000030994 | 182 |
| Left postcentral gyrus, BA 22 | -66,-18,14 | -1.205 | 0.000067115 | 87 |
| Right thalamus | 16,-28,10 | -1.206 | 0.000030994 | 60 |
| **FC** |  |  |  |  |
| FC > HC |  |  |  |  |
| Left superior frontal gyrus, orbital part, BA 11 | -12,30,-22 | 1.303 | 0.000005186 | 443 |
| FC < HC |  |  |  |  |
| Left anterior cingulate / paracingulate gyri | 0,32,22 | -2.353 | ~0 | 1434 |
| Left precuneus, BA 23 | -4,-60,20 | -1.370 | 0.001532733 | 85 |
| Left caudate nucleus, BA 25 | -6,18,-6 | -1.299 | 0.002188206 | 46 |
| Left caudate nucleus | -14,12,18 | -1.301 | 0.002105594 | 21 |
| Left median cingulate / paracingulate gyri, BA 24 | -8,6,40 | -1.299 | 0.002152085 | 11 |

^*^All voxels with *P* < 0.005 uncorrected.

BA: Brodmann area; FC: functional constipation; FD: functional dyspepsia; HC: healthy controls; IBS: irritable bowel syndrome; MNI: Montreal Neurological Institute; SDM: seed‐based *d* mapping.
